# Liposome bupivacaine for pain control after total knee arthroplasty: a meta-analysis

**DOI:** 10.1186/s13018-016-0420-z

**Published:** 2016-07-22

**Authors:** Zhong Qing Wu, Ji Kang Min, Dan Wang, Yong Jian Yuan, Heng Li

**Affiliations:** Department of Orthopedics, The First People’s Hospital of Huzhou, 158 Guangchang hou road, Huzhou, Zhejiang Province 313000 China

**Keywords:** Liposome bupivacaine, Total knee arthroplasty, Pain control, Meta-analysis

## Abstract

**Background:**

Total knee arthroplasty (TKA) is associated with intense and long-duration pain. Research is currently being conducted on the use of liposome bupivacaine (LB) to prolong the effects of local infiltration anesthesia. This systematic review and meta-analysis of randomized controlled trials (RCTs) evaluated the efficacy and safety of pain control of using LB versus placebo after TKA.

**Methods:**

In April 2016, the Medline, Embase, PubMed, Cochrane Controlled Trials Register (CENTRAL), Web of Science, Google database, and Chinese Wanfang databases were searched to identify articles that compare a LB group versus a control group for pain control after TKA. This systematic review and meta-analysis were performed according to the PRISMA statement criteria. The primary endpoint was the visual analogue scale (VAS) score after TKA at 24, 48, and 72 h. The second outcome was nausea complications, which represent morphine-related side effects. Stata 12.0 software was used for the meta-analysis.

**Results:**

Five studies involving 574 patients met the inclusion criteria. The meta-analysis revealed that LB can decrease the VAS score at 24 h (mean difference (MD) = −0.50; 95 % CI −0.97 to −0.04; *P* = 0.034), 48 h (MD = −0.26; 95 % CI −0.71 to 0.19; *P* = 0.256), and 72 h (MD = −0.26; 95 % CI −0.71 to 0.19; *P* = 0.256). There was no significant difference with respect to the length of hospital stay (MD = −0.08; 95 % CI −0.28 to 0.13; *P* = 0.475). Furthermore, LB can reduce the occurrence of nausea (RR = 0.38; 95 % CI 0.18 to 0.79; *P* = 0.009).

**Conclusions:**

Based on the current meta-analysis, LB as a novel anesthetic formulation administration following TKA demonstrated better pain control; however, the sample size was limited, and further RCTs are needed to identify the effects of LB after TKA.

## Background

Total knee arthroplasty (TKA) is a common surgical procedure that causes moderate to severe pain and is considered a challenge to manage due to its intensity and duration [1, 2]. Inadequate pain control after TKA is associated with poor postoperative recovery, an increase in the length of hospital stay, and higher patient dissatisfaction and hospital costs [3, 4]. Many analgesia techniques, such as femoral nerve block, epidural infusions, oral morphine, peri-articular infiltration analgesia, and adductor canal block, have been used to control pain after TKA [5, 6]. Multimodal analgesia including local infiltration analgesia has been identified as the most preferred method for pain control after TKA [7]. These methods not only reduce the pain intensity but also decrease opioid consumption and thereby reduce the occurrence of the morphine-related complications such as nausea, vomiting, and respiration depression [8].

Bupivacaine HCL has been used in many surgical procedures as a local anesthetic due to its long-acting effects. However, even with the addition of epinephrine to prolong the duration of the analgesic effects, these effects remain limited [9]. Liposome bupivacaine (LB) is a novel local anesthetic that enables the extended release of bupivacaine into surgical sites to extend analgesic effects. The mechanism of LB to extend analgesia effects uses DepoFoam® as a delivery platform; thus, bupivacaine can be released over 72 h [10]. LB has been used to control pain after TKA; however, there is no consensus regarding the pain control effects of LB after TKA. Thus, a systematic review and meta-analysis was conducted to further analyze the effects of LB for pain control after TKA.

## Methods

### Search strategies

A comprehensive search for all relevant studies comparing LB with control after primary TKA was conducted using the electronic databases PubMed, Embase, the Cochrane Library, and Wanfang database from their inception until April 2016. Relevant studies were also manually searched in reviews and gray literature, and this process was halted when no additional articles could be added. The search terms were as follows: “total knee arthroplasty,” “total knee replacement,” “TKA,” “TKR,” “Arthroplasty, Replacement, Knee[Mesh],” and “liposome bupivacaine.” These key words and the corresponding medical subject heading (MeSH) terms were combined with the Boolean operators AND and OR. There were no restrictions regarding the language or year of publication.

### Inclusion criteria and study selection

All randomized controlled trials (RCTs) comparing LB with control were eligible for this meta-analysis. If there was more than one eligible trial from one team, the study with the most recent publication data was enrolled for analysis. Studies with bilateral TKA, TKA revision, or administration with other anesthetic methods were excluded. All non-randomized trials were also excluded.

### Data extraction

Two reviewers extracted data independently using a predefined data extraction form. Disagreements were resolved by discussion or consensus with a third reviewer. The data extracted included the first author, publication years, study characteristics (number of patients and percentage of female patients), participant characteristics (i.e., mean age, anesthesia, operative approach, and type of prosthesis), and the length of follow-up. For studies with insufficient information, the reviewers attempted to contact the first author via e-mail or telephone to obtain the original data. After duplicates were excluded, two reviewers independently read the titles and abstracts of the searched literature. Most of the articles were excluded based on the topic of the article provided in the respective title or abstract, and disagreements regarding whether an article should be included were resolved in discussion or by a senior reviewer. Postoperative pain intensity was measured using a 100-point visual analogue scale (VAS). The 10-point VAS score was converted to a 100-point VAS score. Data in other forms (i.e., median, interquartile range, and mean ± 95 % CI) were converted to means ± SD according to the Cochrane Handbook. If the data were not reported numerically, we extracted them using the GetData Graph Digitizer software from the published figures.

### Quality assessment

Two independent reviewers assessed the methodological quality of the included trials according to the Cochrane Collaboration recommendations. The following information was evaluated: random sequence generation, allocation concealment, blinding of outcome assessments, incomplete outcome data, selective reporting, and other biases. An independent arbiter was consulted to reconcile any disagreements.

### Statistical analysis

Continuous outcomes, such as the VAS at 24, 48, and 72 h and the length of hospital stay, were expressed as the mean difference (MD) with respective 95 % CIs. Discontinuous outcomes (the rate of nausea) were expressed as the relative risk (RR) with 95 % CIs. Statistical significance was set at *P* < 0.05 to summarize findings across the trials. Software Stata 12.0 (Stata Corp., College Station, TX) was used for the meta-analysis. Statistical heterogeneity was tested using the chi-squared test and *I*^2^ statistic. A chi-squared test *I*^2^ > 50 % was considered suggestive of statistical heterogeneity; thus, a random effects model was adopted and further subgroup analysis or sensitivity analysis was conducted to seek out the potential source of heterogeneity. When there was no statistical evidence of heterogeneity, a fixed effect model was adopted.

## Results

### Search results

**Fig. 1:**
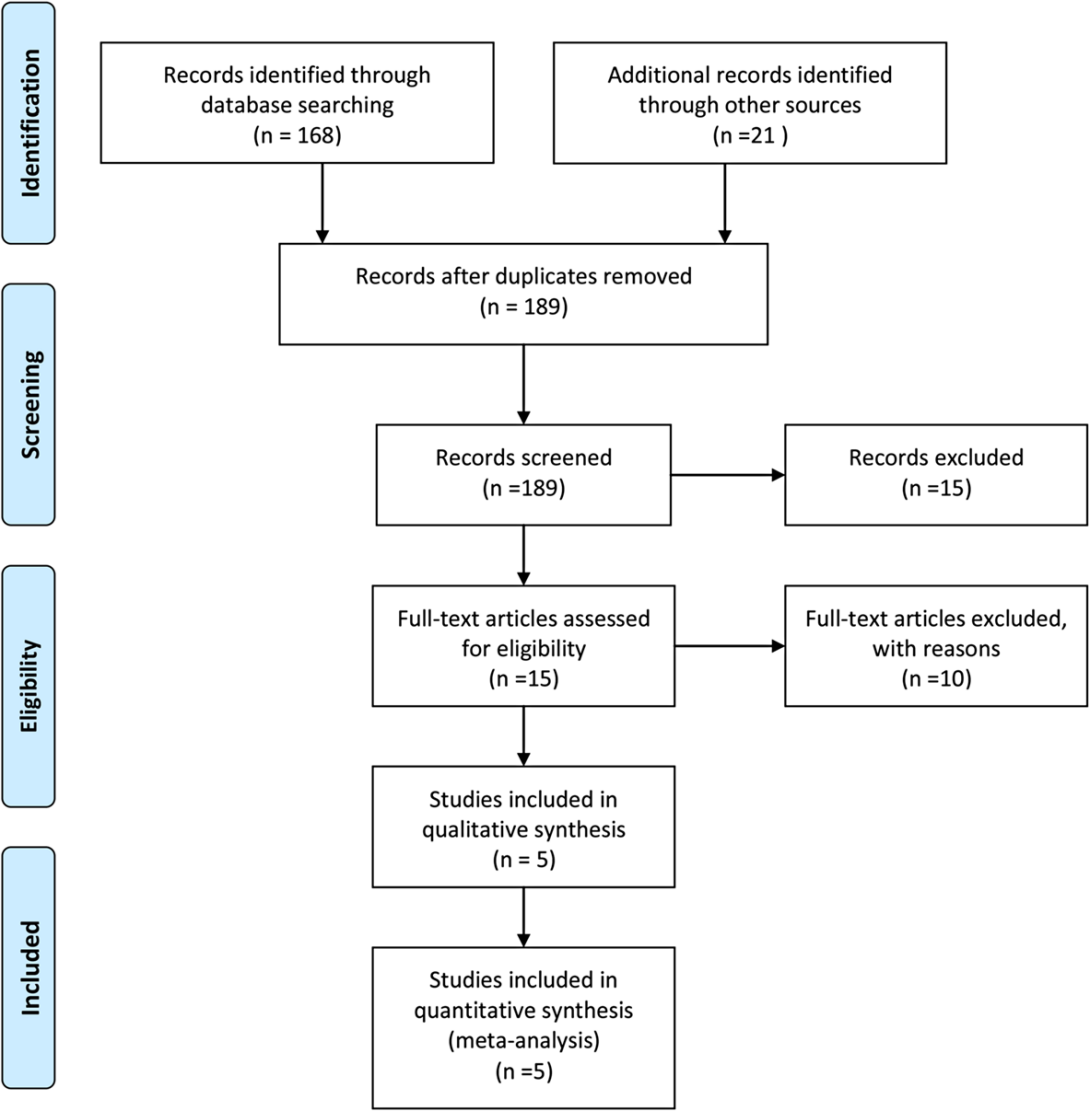
The search strategies of the included studies

In the initial research, 125 potentially relevant trials were identified and 32 duplications were removed using the software Endnote X7 (Thomson Research Soft Company, America). Finally, the title and abstract of the 93 articles were reviewed and included according to the inclusion criteria (detailed search criteria can be found in Fig. 1). During the search process, a total of three disagreements occurred; these were resolved by the senior author following review of the full article. Finally, only five RCTs were included for the meta-analysis [11–15]. One study compared four different doses of LB with a control group; this study was divided into four trials [11]. Thus, eight studies with a total of 574 patients involving 276 LB patients and 298 control patients were finally included in this meta-analysis. All selected patients were in English and published between 2012 and 2016. The detailed characteristics of the included studies are shown in Table 1.

**Table 1:** The general characteristic of the included studies

Author	Country	No. of patients			Female (%)		Age (mean)		Anesthesia	Approach	Use of cement	Indication of pain	Follow-up (month)
		Total	LB	CO	LB	CO	LB	CO					
Bramlett 2012 [11] 133mg	USA	62	28	34	53.6	67.6	61.4	62.2	General anesthesia	NS	NS	NRS	1
Bramlett 2012 [11] 266mg	USA	59	25	34	48	67.6	61.1	62.2	General anesthesia	NS	NS	NRS	1
Bramlett 2012 [11] 399mg	USA	60	26	34	57.7	67.6	61.8	62.2	General anesthesia	NS	NS	NRS	1
Bramlett 2012 [11] 532mg	USA	59	25	34	80	67.6	64.9	62.2	General anesthesia	NS	NS	NRS	1
Collis 2016 [15]	USA	105	54	51	53.7	72.5	63	63	General anesthesia	Subvastus approach	NS	VAS	NS
Schroer 2015 [12]	USA	111	58	53	59	60	67	68.6	Spinal or general anesthesia	Mini-subvastus approach	Cemented	VAS	0.75
Schwarzkopf 2016 [13]	USA	38	20	18	67	43	63	59	Spinal anesthesia	Medial parapatellar approach	Cemented	VAS	NS
Surdam 2015 [14]	USA	80	40	40	57.5	52.5	64.9	68.4	Spinal anesthesia	NS	Cemented	Patient self-rated 0–10 pain scale and the Wong–Baker pain faces scale	10

NRS, numeric rating score; NS, not stating, LB, liposome bupivacaine; CO, control

### VAS score at 24 h

**Fig. 2:**
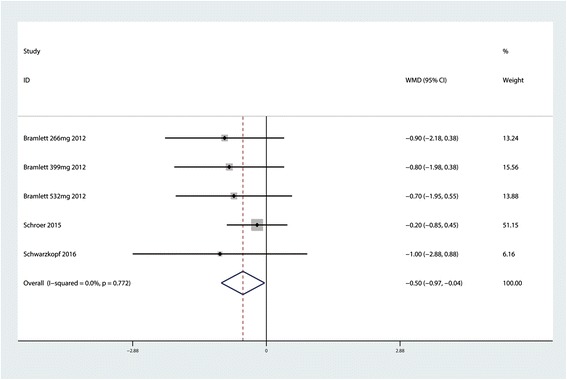
Forest plot of comparison: VAS score at 24 h between the LB group and control group

Five trials reported a VAS score at 24 h for the LB group and control group. Pooled results indicated that LB was associated with a lower VAS score at 24 h after TKA (MD = −0.50; 95 % CI −0.97 to −0.04; *P* = 0.034, Fig. 2) with no heterogeneity (*P* = 0.072, *I*^2^ = 0.0 %).

### VAS score at 48 h

**Fig. 3:**
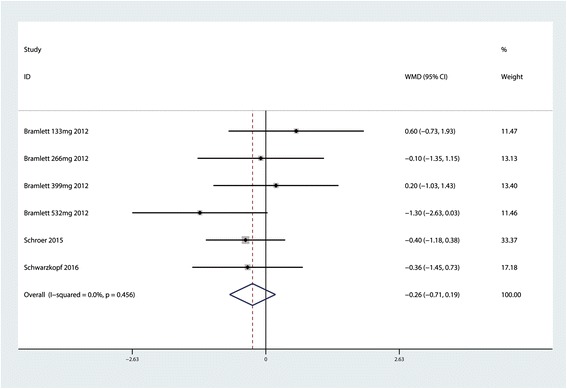
Forest plot comparing VAS score at 48 h between the LB group and control group

Pooled results indicated no significant difference between the LB and control group after TKA in terms of VAS score at 48 h (MD = −0.26; 95 % CI −0.71 to 0.19; *P* = 0.256, Fig. 3) with no heterogeneity (*P* = 0.456, *I*^2^ = 0.0 %).

### VAS score at 72 h

**Fig. 4:**
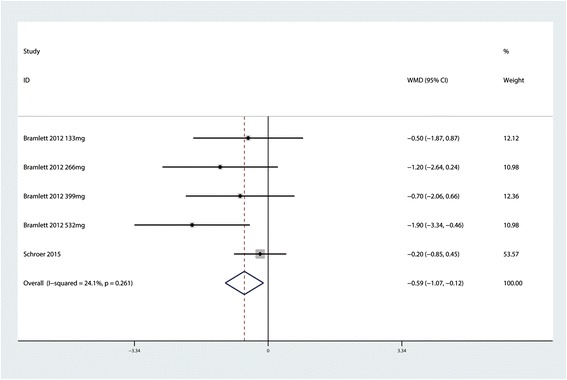
Forest plot comparing VAS score at 72 h between the LB group and control group

Five trials consisting of 354 patients reported the VAS score at 72 h for the LB group and control group. The meta-analysis indicated that peri-articular infiltration LB can decrease the VAS score at 72 h (MD = −0.26; 95 % CI −0.71 to 0.19; *P* = 0.256, Fig. 4) with low heterogeneity (*P* = 0.261, *I*^2^ = 24.1 %). A sensitivity analysis after excluding one trial with no heterogeneity demonstrated a significant difference between the two groups (MD = −0.26; 95 % CI −1.75 to 0.35; *P* = 0.003), a result that is in line with previous analysis.

### Length of hospital stay

**Fig. 5:**
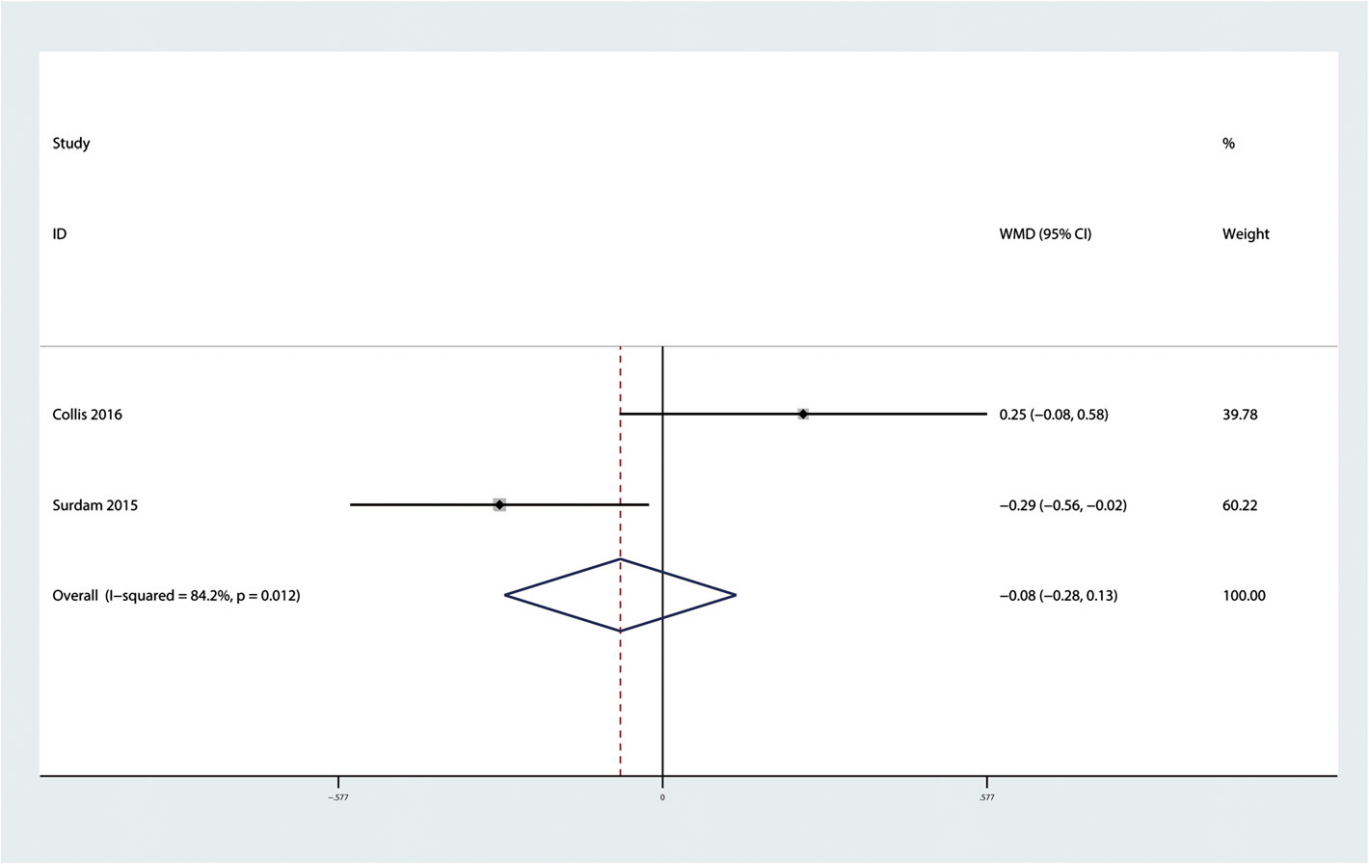
Forest plot comparing the length of hospital stay between the LB group and control group

Two studies reported the length of hospital stay in the two groups. There was no significant difference between the LB and control group in terms of the length of hospital stay (MD = −0.08; 95 % CI −0.28 to 0.13; *P* = 0.475, Fig. 5) with a high heterogeneity (*P* = 0.475, *I*^2^ = 84.2 %). However, because there were only two trials, a sensitivity analysis was not conducted.

### The occurrence of nausea

**Fig. 6:**
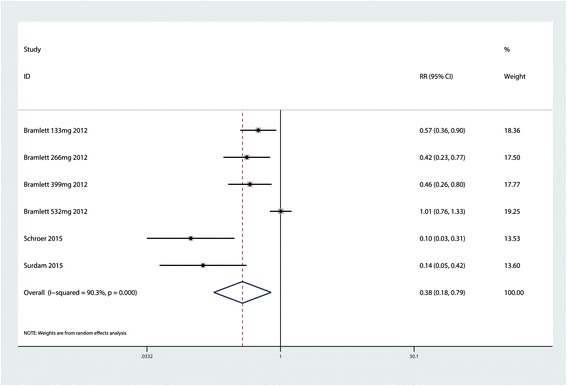
Forest plot comparing the occurrence of nausea between the LB group and control group

The meta-analysis indicated that LB can decrease the occurrence of nausea (RR = 0.38; 95 % CI 0.18 to 0.79; *P* = 0.009, Fig. 6) with a high heterogeneity (*P* = 0.000, *I*^2^ = 90.3 %). A random effects model was thus adopted to further analyze the results. Furthermore, a sensitivity analysis was conducted to determine which study contributed to the final large heterogeneity; however, the sensitivity analysis indicated that no trials influenced the final heterogeneity.

## Discussion

To our knowledge, this is the first systematic review and meta-analysis of RCTs comparing LB with a control in the management of pain after TKA. Only RCTs of high quality were included for the meta-analysis, which strengthens the credibility of our results. This comparative and retrospective study presents selective risk bias regarding the final results. All of the included studies were published after 2012, and most trials were published in 2015 and 2016. Of all the included studies, only one trial was randomized via random sequence generation, and one study performed allocation using sealed envelopes. Three studies were double-blinded to the participants and patients. All of the studies completed the outcome data, and selective reporting and other biases are not clear. Detailed information on the included studies can be found in Table 2. Pain intensity was measured as VAS score at 24, 48, and 72 h after TKA, and the results indicated that peri-articular administration LB can decrease the VAS score at 24, 48, and 72 h. However, there is no significant difference between the two groups in terms of VAS score at 48 h. There are insufficient data regarding long-term pain control of LB with placebo in the included studies; long-term follow-ups are needed. Regarding the length of hospital stay, because a total of two studies reported relevant data, there was no significant difference between the two groups. Furthermore, LB can also decrease the occurrence of nausea with statistical significance.

**Table 2:** The quality assessment for the included studies

Trials	Random sequence generation	Allocation concealment	Blinding	Complete outcome data	Selective reporting	Other bias
Bramlett 2012 [11]	Centralized randomization system	Unclear	Yes	Yes	Unclear	Unclear
Collis 2016 [15]	Unclear	Unclear	Yes	Yes	No	Unclear
Schroer 2015 [12]	Unclear	Unclear	Yes	Yes	Unclear	Unclear
Schwarzkopf 2016 [13]	Unclear	Unclear	Unclear	Yes	Unclear	Unclear
Surdam 2015 [14]	Unclear	Sealed envelopes	No	Yes	Unclear	Unclear

LB was recently introduced to multiple anesthesia with the intention of improving the analgesic effects of peri-articular infiltration (PAI) anesthesia by increasing the duration and consistency of action. However, recent studies support indeterminate efficacy of LB versus PAI. In this meta-analysis, LB can decrease the pain intensity at 24, 48, and 72 h after TKA. Cien et al. [16] compared LB versus femoral nerve block after TKA and found that LB can reduce hospital costs and opioid consumption. Liposomes are commonly used drug carriers that offer biologically active drugs with long-term potency and a reduction in toxicity [17]. LB consists of microscopic, spherical multi-vesicular lipid-based particles that form honeycomb-like aqueous chambers. This structure combined with bupivacaine enables the diffusion of bupivacaine in the knee joints over 96 h after a single administration. As a natural evolution, LB is now being proposed to offer benefits in TKA [18]. However, when comparing PAI in LB with a multimodal pain management protocol with bupivacaine, epinephrine, and morphine, the pain score in the LB group is higher than that in the control group [10]. Thus, solely peri-articular LB is not sufficient to achieve the most ideal anesthetic effect.

LB can lead to shortened hospital stay; however, this difference was not statistically significant because the samples and included trials were limited and the length of hospital stay is affected by many factors. Chughtai et al. [19] used a large hospital database to determine whether LB can reduce hospital stay length and found that the length of hospital stay (LOS) in the LB group was 2.58 days; this length was 2.98 days in the non-injection group. In contrast, Schroer et al. [12] found a benefit for multimodal LB suspension injection versus bupivacaine HCL; however, there were no significant differences between the pain score at 24, 48, and 72 h. Furthermore, the cost of LB was higher than that of the control group, so the authors did not recommend routine LB. However, a statistically significant difference may have not been achieved because the sample size may have not allowed for adequate power. Additionally, Schroer et al. [12] did not determine the LOS or discharge outcome. The difference in cost may be balanced by the shorter LOS in this meta-analysis.

Nausea and vomiting are common complications that usually occur following oral or intravenous morphine. Multimodal anesthetic techniques can reduce the consumption of morphine and subsequent morphine-related complications. If patients have better pain control with local infiltration analgesia, the consumption of morphine will decrease correspondingly. Our meta-analysis indicated that local infiltration with LB can also reduce nausea.

There are several limitations to this meta-analysis: (1) only five RCTs were included, and the sample sizes in each trial were small, which would affect the final results; (2) the duration of follow-up in some studies was unclear, and long-term follow-up was needed for this analysis; (3) the publication bias of the meta-analysis also influenced the results; and (4) the dose of LB and the randomized generation sequence were not reported in some trials.

## Conclusions

In conclusion, this was the first systematic review to evaluate the efficacy and safety of using LB compared with placebo for reducing pain after TKA. Our meta-analysis indicates that LB may be more effective in pain control at 24, 48, and 72 h compared with using a placebo after TKA. The most important finding of this study is that LB may shorten the hospital stay and reduce the occurrence of nausea. In future research, an optimal dose of LB should be rigorously defined, and the perioperative multimodal anesthesia should also be clarified. More importantly, well-designed trials with larger sample sizes are needed to provide further reliable evidence for the safety of LB for pain management, knee flexion, and other outcomes after TKA.

## Abbreviations

CENTRAL, Cochrane Controlled Trials Register; LB, liposome bupivacaine; LOS, length of hospital stay; MD, mean difference; MeSH, medical subject heading; PAI, peri-articular infiltration; RCT, randomized controlled trials; RR, relative risk; TKA, total knee arthroplasty; VAS, visual analogue scale
